# Corrosion Behavior and Mechanism of 304 Stainless Steel Welded Joints in Simulated Freshwater Environment

**DOI:** 10.3390/ma18133074

**Published:** 2025-06-28

**Authors:** Yue Yu, Xiayan Wang, Shilong Wei, Zengyao Chen, Zhanhua Wang, Mengnan Li, Zhiyong Liu

**Affiliations:** 1 Zhengzhou Yellow River Hydro Power Development General Company, Zhengzhou 459000, China; 2 Corrosion and Protection Center, University of Science and Technology Beijing, Beijing 100083, China; 3 Shuigong Metal Structure Quality Inspection Testing Center, Ministry of Water Resources, Zhengzhou 453400, China

**Keywords:** 304 stainless steel, welded joint, freshwater environment, corrosion resistance

## Abstract

This investigation examines the corrosion behavior and mechanisms of 304 stainless steel shielded metal arc welding (SMAW) and gas metal arc welding (GMAW) joints in the simulated reservoir environment through electrochemical testing, stress-free hanging specimens and U-bend specimen immersion experiments, and microstructural characterization. The electrochemical results demonstrate that both welded joints exhibit a superior corrosion resistance in this environment, with a sensitivity of intergranular corrosion (IGC) below 1% and a corrosion rate below 0.005 mm/a. Increasing chloride concentrations elevate the passivation current densities for both the base metal and welded joints. The immersion testing revealed that after 90 days of exposure across the investigated chloride concentrations (50–300 mg/L), all welded specimens maintained surface integrity with no visible corrosion. Furthermore, U-bend specimens demonstrated no evidence of stress corrosion cracking, confirming a low stress corrosion susceptibility.

## 1. Introduction

Stainless steel is extensively employed in water conservancy infrastructure as piping systems, valves, and other critical components due to its superior mechanical properties and corrosion resistance [1]. However, the electrochemical heterogeneity resulting from compositional [2] and microstructural heterogeneities [3] between the welded joints and base metal and residual stresses from the welding process [4] may induce an elevated susceptibility to localized corrosion mechanisms, including pitting [5] and stress corrosion cracking (SCC) [6,7], ultimately inducing structural degradation through perforations or fractures [8]. Consequently, it is imperative to systematically evaluate and predict the corrosion behavior of stainless steel welded joints under operational conditions.

The corrosion behavior of stainless steel welded joints has been widely investigated in both academic studies and industrial applications. A notable case involved a 304 stainless steel piping system at a machinery manufacturing plant where fabricated components were prematurely cracking at the welds connecting to matching flanges. A metallurgical analysis [9] revealed chromium depletion in the heat-affected zone (HAZ) accompanied by subsequent oxide precipitation along grain boundaries, ultimately initiating intergranular corrosion and fracture. Ryl et al. [10] corroborated these findings through a systematic investigation of localized corrosion in water distribution systems, identifying an improper welding parameter selection and associated microstructural alterations as primary causative factors.

Garcia et al. quantitatively evaluated pitting [11] and intergranular corrosion (IGC) [12] susceptibilities across weld zones using electrochemical potentiokinetic reactivation (EPR) and double-loop EPR (DL-EPR) methods, establishing process-dependent correlations and confirming the HAZ as the most corrosion-vulnerable region. In dissimilar welded joint research, Wang et al. [13] fabricated 2205 duplex stainless steel/16MnR joints via shielded metal arc welding (SMAW) and gas metal arc welding (GMAW), with a comparative analysis demonstrating GMAW’s marginally superior corrosion resistance. Bansod et al. [14] systematically examined thermal processing parameters, revealing that an excessive heat input induces a significant reduction in the δ-ferrite phase fraction, consequently impairing the pitting resistance. A similar conclusion was also reached by Gucwa et al. [15]. Ge et al. [16] investigated the effect of different arc energies on the microstructure and corrosion behavior of ER2209 duplex stainless steel deposited by GMAW on Q345B low-alloy steel and found that the microstructure and elemental distribution of the welded joints are significantly affected by the arc energy, which in turn affects their corrosion performance. Cho et al. [17] compared the corrosion behavior of super duplex stainless steel welded tubes under different welding processes, pointing out that a lower heat input and higher cooling rate are the causes of the good corrosion resistance of welded joints. Some studies further demonstrate that an elevated current intensity during welding notably enhances the susceptibility to pitting [18], IGC [19], and SCC [20] in neutral chloride-containing environments.

Beyond weld process parameters and joint quality, the environmental chloride ion concentration plays a critical role in governing the corrosion performance of stainless steel welded joints. Ma et al. [21] demonstrated an inverse correlation between the SCC susceptibility and both the temperature and chloride concentration in 304 stainless steel joints. Through an electrochemical investigation and a nonlinear Mott–Schottky analysis method of 304 stainless steel in solutions with different pH values and chloride concentrations, Gao et al. [22] revealed that the chloride-induced cation vacancy accumulation in 304 stainless steel’s passive film leads to an increasing pitting susceptibility, culminating in a catastrophic passive film breakdown and pit nucleation. Furthermore, Zhang et al. [23] identified nanoscale structural heterointerfaces between crystalline and amorphous domains within passive films, which serve as preferential diffusion pathways for chloride ion ingress.

However, the operational performance of 304 stainless steel welded joints in freshwater environments remains insufficiently characterized. Field investigations at a certain reservoir revealed pervasive pitting corrosion at 304 stainless steel weldments, with a critical wall thickness reduction observed in water-conveyance pressure conduits. Concurrently, carbon steel components in irrigation tunnels exhibited a service degradation with maximum thickness loss rates approaching 40%, though fundamental degradation mechanisms remain poorly understood.

Shielded metal arc welding (SMAW) is a manual process using a consumable electrode and workpiece as opposing terminals, where the arc heat melts and joins base metals. Gas metal arc welding (GMAW) employs a continuously fed wire electrode to generate an arc that simultaneously melts filler and base metals under a protective gas environment (e.g., CO_2_/Ar mixtures), which prevents the atmospheric contamination of the molten metal. Both techniques are extensively utilized in hydraulic engineering for their field adaptability and reliable joint durability. This study systematically examines pipeline corrosion cases in a freshwater environment, focusing on 304 stainless steel weld microstructures. Through a comparative evaluation of SMAW and GMAW joints under controlled chloride concentrations (50–300 mg/L), we investigated corrosion resistance variations and elucidate fundamental mechanisms governing localized degradation in freshwater environments.

## 2. Materials and Methods

**Figure 1:**
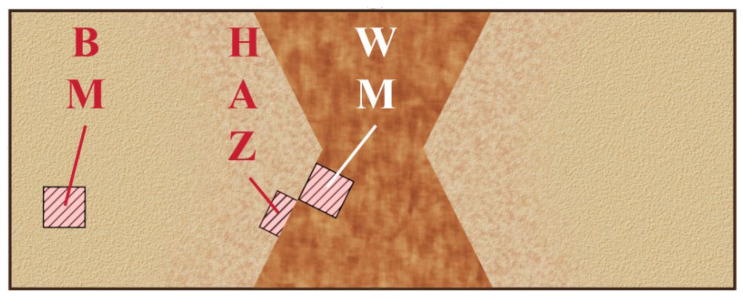
The sampling location at the welded joint.

The selected material is 304 austenitic stainless steel with a composition of C 0.02%, Si 0.38%, Mn 1.04%, P 0.037, S 0.002%, Cr 18.3%, Ni 8.02%, N 0.07%, and the remainder being Fe (Table 1). Parameter-optimized welded joints were fabricated via SMAW (110–120 A DC+, A102 electrode) and GMAW (24–28 V/180–220 ipm, ER308 wire with 98%Ar + 2%CO_2_ shielding gas). The chemical compositions of the welding feeding wire are shown in Table 1. In this experiment, samples of the base material (BM), weld metal zone (WM), and HAZ were wire cut from positions shown in Figure 1. The dimensions of the samples from the WM and BM area were 10 mm × 10 mm × 4 mm, while HAZ samples were constrained to 10 mm × 5 mm × 2 mm to maintain microstructural uniformity. All specimens underwent sequential grinding with SiC papers (400–3000 grit), final polishing with a 1.5 μm diamond suspension, followed by ultrasonic cleaning in deionized water for 30 s and ethanol for 180 s, and then were dried with cold air. Prior to the microstructural characterization, specimens were chemically etched in mixed acid solution (HCl:HNO_3_ = 3:1 vol%) for 15–25 s at 25 ± 2 °C. To compare the microstructural differences in welded joints prepared by different welding processes, the metallographic structures of BM, WM, and HAZ samples were then observed using a KEYENCE VHX-2000 (Shanghai, China) optical microscopy (OM).

Through an actual investigation of a certain reservoir, the main ions present in the reservoir environment and their concentrations are as follows: Cl^−^ 101.30 mg/L, HCO_3_^−^ 190.70 mg/L, SO_4_^2−^ 130.00 mg/L, Na^+^ 166.58 mg/L, Ca^2+^ 10.56 mg/L, Mg^2+^ 9.59 mg/L, and K^+^ 3.14 mg/L. Electrolyte solutions were formulated to simulate the multi-ion composition of the freshwater environment, with chloride concentration gradients of 50, 100, 150, and 300 mg/L, the detailed composition is provided in Table 2.

All electrochemical and immersion experiments were conducted in a controlled temperature environment (25.0 ± 0.5) °C using BM, WM, and HAZ specimens. Electrochemical measurements were carried out through a Princeton Applied Research PARSTAT 2273 electrochemical workstation (Oak Ridge, TN, USA), employing a standard three-electrode configuration with a saturated calomel reference electrode (SCE) as the reference electrode, a platinum electrode as the counter electrode, and the samples as the working electrode. Prior to electrochemical testing, all samples were subjected to a constant potential polarization at −1.5 V (vs. SCE) for 3 min to remove the oxide layer from the surface; this was immediately followed by an open-circuit potential test for about 30 min to allow for potential stabilization. Electrochemical impedance spectroscopy (EIS) tests were performed at frequencies from 100 kHz to 10 mHz and an AC voltage of a 10 mV amplitude, and data were fitted using ZSimpwin 3.60 software. The kinetic potential polarization test was set up with a scan rate of 0.5 mV/s and a scan range of −0.5 to 1.5 V (vs. SCE). For the IGC assessment, DL-EPR curves were scanned from 100 mV (vs. OCP) to 350 mV (vs. SCE) with a rate of 1.667 mV/s performed in a freshly prepared 0.5 M H_2_SO_4_ + 0.02 M KSCN solution.

**Figure 2:**
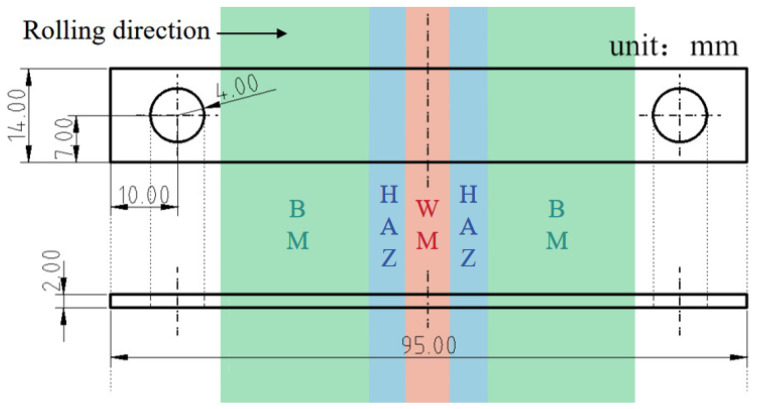
Schematic diagram of U-bend specimen size.

Stress-free hanging specimens (BM, SMAW, and GMAW joints) underwent a 90-day immersion in simulated solutions with chloride concentrations of 100 mg/L and 300 mg/L (Table 2) to evaluate the service-relevant corrosion performance. For the stress corrosion assessment, U-bend samples were prepared from the transverse direction of the welded plate, with the length direction parallel to the rolling direction, as shown in Figure 2. All SCC tests maintained identical environmental parameters (solution chemistry and temperature) and exposure durations as static immersion experiments.

Following the immersion exposure, corrosion products on the hanging specimens and U-bend samples were chemically removed. The chemical descaling solution was formulated per GB/T 16545 [24]: 500 mL HCl (37 vol%) and diluted to 1000 mL with deionized water (final concentration 18.5 vol%), with 0.35 wt% hexamethylenetetramine (C_6_H_12_N_4_) added as a corrosion inhibitor. The mass loss was determined using an analytical balance with triplicate measurements. The corrosion rate was calculated according to Formula (1) [25]:

(1) $$V=\frac{8.76(W_{0}-W_{1})}{St\rho }$$

In the equation, *V* (mm/a) represents the corrosion rate; *W*_0_ and *W*_1_ (g) represent the initial and final masses; *S* (m^2^) represents the exposed sample surface area; *t* (h) represents the exposure duration; and *ρ* (g/cm^3^) is the sample density (7.93 for 304 SS).

The microstructure of the descaled samples was observed using optical microscopy (OM) and scanning electron microscopy (SEM). The U-bend samples were examined for stress corrosion.

## 3. Results

### 3.1. Metallographic Structure Observation

**Figure 3:**
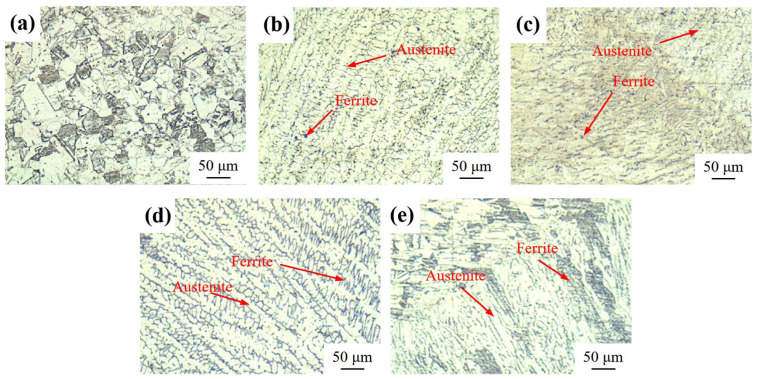
Metallographic structure of 304 stainless steel BM and different weldments. (**a**) BM; (**b**) SMAW-WM; (**c**) SMAW-HAZ; (**d**) GMAW-WM; and (**e**) GMAW-HAZ.

Figure 3 compares the microstructural evolution of the 304 stainless steel BM, WM, and HAZ under SMAW and GMAW processes. Optical microscopy reveals that the structure of the 304 stainless steel BM is austenite, while the metallographic microstructures of the WM in SMAW and GMAW (Figure 3b,d) are both dual-phase δ-ferrite/austenite microstructures; in the metallographic photos of the HAZ (Figure 3c,e), ferrite is distributed along the austenite grain boundaries. No inclusions were observed in any of the metallographic photos, indicating a good preparation quality of both the 304 stainless steel BM and the welded joint samples. When austenitic stainless steel contains a certain amount of ferrite with a good toughness, it can improve the intergranular corrosion and stress corrosion resistance of the WM [26]. Although the content and distribution uniformity of the ferrite observed in the SMAW-WM structure are lower than those in the GMAW-WM structure, the grain size of the structure obtained by GMAW is slightly larger in the HAZ than that of the SMAW, and the size difference between grains in the HAZ is larger, which may reduce the corrosion resistance of the structure obtained by GMAW.

### 3.2. Corrosion Test Results

#### 3.2.1. Dynamic Potential Reactivation Curve Test Results

**Figure 4:**
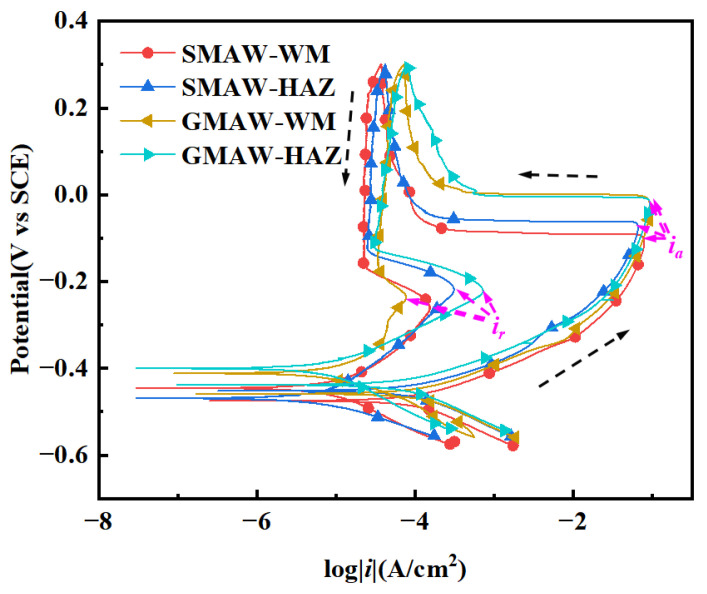
Dynamic potential reactivation curve of different 304 stainless steel welding specimens.

Figure 4 presents the DL-EPR curves for 304 stainless steel weldment regions (WM/HAZ). The activation current density peak (*i_a_*) of the GMAW joint microstructure is relatively higher, reflecting an enhanced corrosion tendency for the sensitized specimen. The reactivation current density peak (*i_r_*) originates from the Cr-depleted zone induced by the precipitation of M_23_C_6_. The degree of sensitization (*DOS*) is defined as the following formula [27]:

(2) $$DOS=\frac{i_{r}}{i_{a}}\times 100\%$$

The DOS values are tabulated in Table 3. The analysis reveals a low intergranular corrosion susceptibility across all specimens, with DOS values remaining below 1%. Notably, HAZ regions exhibit generally elevated DOS values compared to WM areas, indicating differential electrochemical properties between weld zones [12].

#### 3.2.2. Electrochemical Test Results

**Figure 5:**
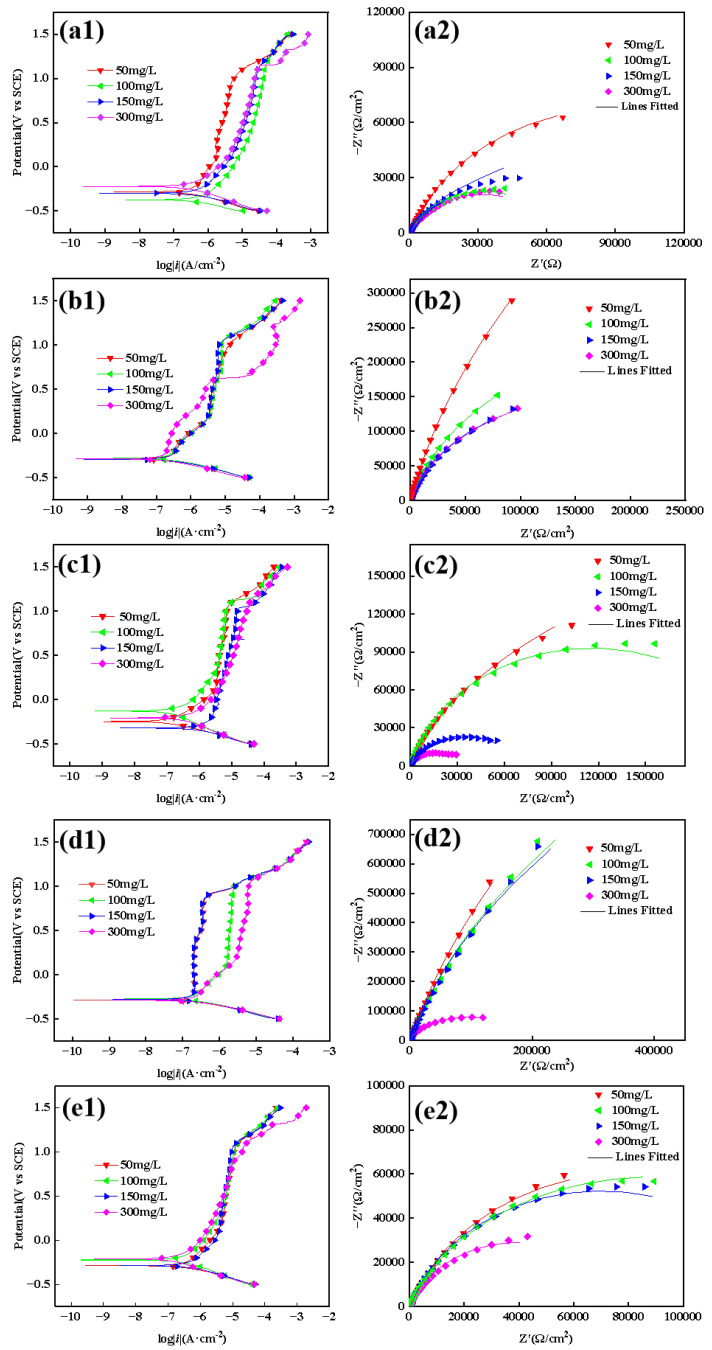
Electrochemical test results at different Cl^−^ concentrations: polarization curve of (**a1**) BM, (**b1**) SMAW-WM, (**c1**) SMAW-HAZ, (**d1**) GMAW-WM, and (**e1**) GMAW-HAZ and Nyquist plot of (**a2**) BM; (**b2**) SMAW-WM; (**c2**) SMAW-HAZ; (**d2**) GMAW-WM; and (**e2**) GMAW-HAZ.

Figure 5 compares the potentiodynamic polarization curves and electrochemical impedance spectroscopy (EIS) of the 304 stainless steel BM versus weld regions (WM/HAZ) in controlled chloride environments. The analysis reveals congruent electrochemical signatures across all specimens: the polarization curves demonstrate passivation characteristics in the anodic region coupled with a dominant oxygen reduction process in the cathodic region. In other words, the corrosion mechanisms of the 304 stainless steel BM and welded joints are the same in the given range of chloride ion concentrations. However, there are differences in the extent to which the chloride ion concentration affects the corrosion resistance of different welded tissues. For example, comparing the HAZ region of the SMAW with that of the GMAW (Figure 5 (c2,e2)), it can be seen that the capacitive arc radius of the HAZ region of the GMAW decreases only slightly when the concentration of chloride ions is elevated, but the capacitive arc radius of the HAZ region of the SMAW decreases more significantly.

**Figure 6:**
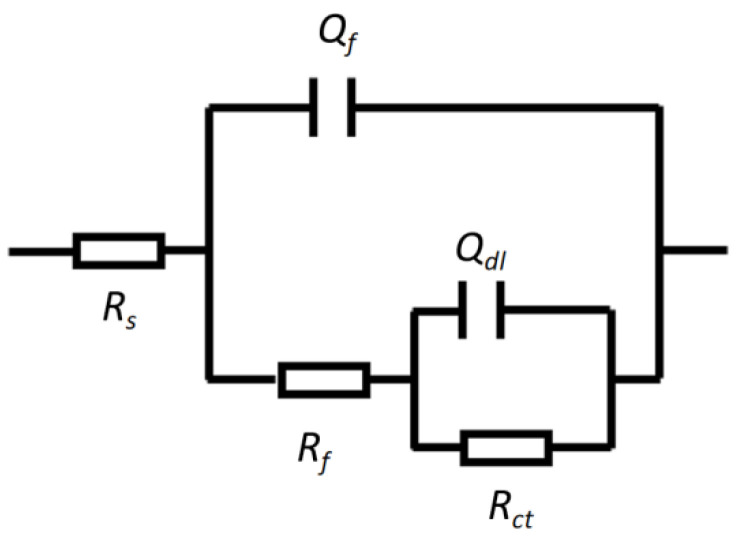
Equivalent circuit used for EIS data fitting.

**Figure 7:**
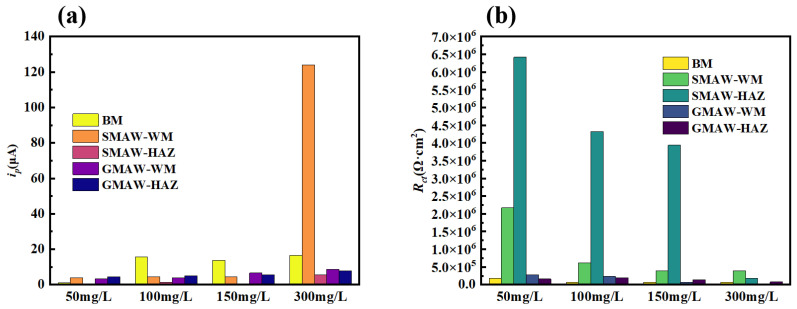
Fitting results of (**a**) *i_p_* and (**b**) *R_ct_* of 304 stainless steel BM and different welded joints at different Cl^−^ concentrations.

To further quantify the electrochemical test results, the measured polarization curves and electrochemical impedance spectra were modeled using the circuit diagram shown in Figure 6. The passive current *i_p_* and charge transfer resistance *R_ct_* of the 304 stainless steel BM and different welded joint structures under various chloride concentrations are listed in Figure 7. Minimum *i_p_* values at 50 mg/L Cl^−^ demonstrate optimal passivation; as the chloride concentration increases, the passive current increases slightly, indicating an acceleration of the corrosion process. In the SMAW-WM area, a significant secondary passivation phenomenon occurs at a chloride concentration of 300 mg/L, and the corresponding passive current density increases significantly, indicating a relatively sharp decrease in the corrosion resistance at this point. From the electrochemical impedance spectra and their fitting results, it can be understood that as the Cl^−^ concentration increases, the impedance arc radius of the 304 stainless steel BM and different welded structures tends to increase, and the charge transfer resistance *R_ct_* decreases, which also indicates a reduction in corrosion resistance. However, the corrosion rate increase across all specimens confirms that the Cl^−^-induced degradation remains within acceptable thresholds.

The comparative analysis of the passive current density (*i_p_*) and charge transfer resistance (*R_ct_*) between SMAW and GMAW joints reveals chloride-dependent corrosion behavior. When the chloride concentration is below 300 mg/L, the SMAW WM and HAZ maintain lower *i_p_* values with corresponding higher *R_ct_* magnitudes than their GMAW counterparts, demonstrating a superior passivation stability; when the chloride concentration reaches 300 mg/L, secondary passivation occurs in the WM area of the SMAW joint, and the passivation current increases significantly. This electrochemical state transition ultimately compromises the SMAW joint’s corrosion resistance relative to the GMAW at elevated chloride levels.

#### 3.2.3. Immersion Test Results

**Figure 8:**
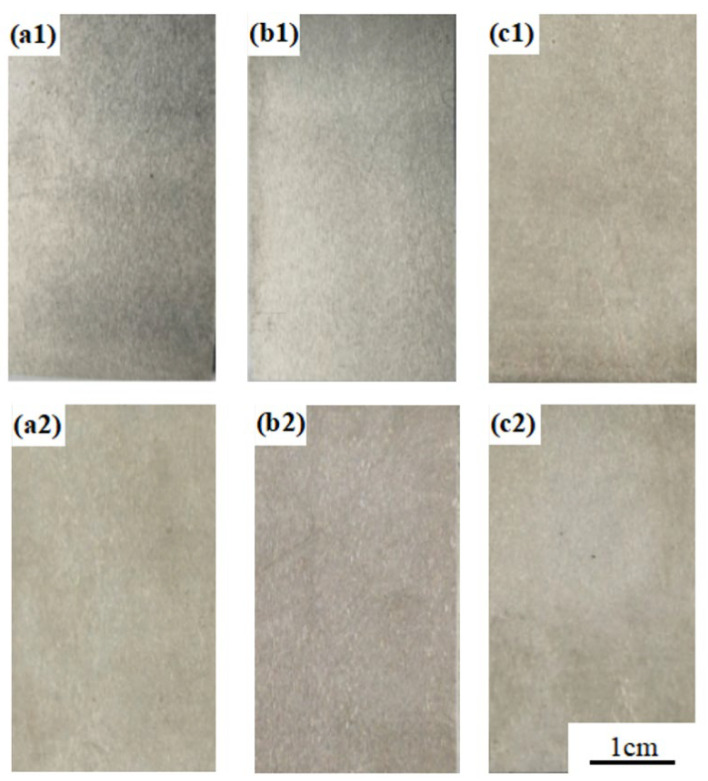
Macroscopic corrosion morphology of immersed specimens in different Cl^−^ environments for 90 days: (**a1**) BM-100 mg/L; (**a2**) BM-300 mg/L; (**b1**) SMAW-100 mg/L; (**b2**) SMAW-300 mg/L; (**c1**) GMAW-100 mg/L; and (**c2**) GMAW-300 mg/L.

**Figure 9:**
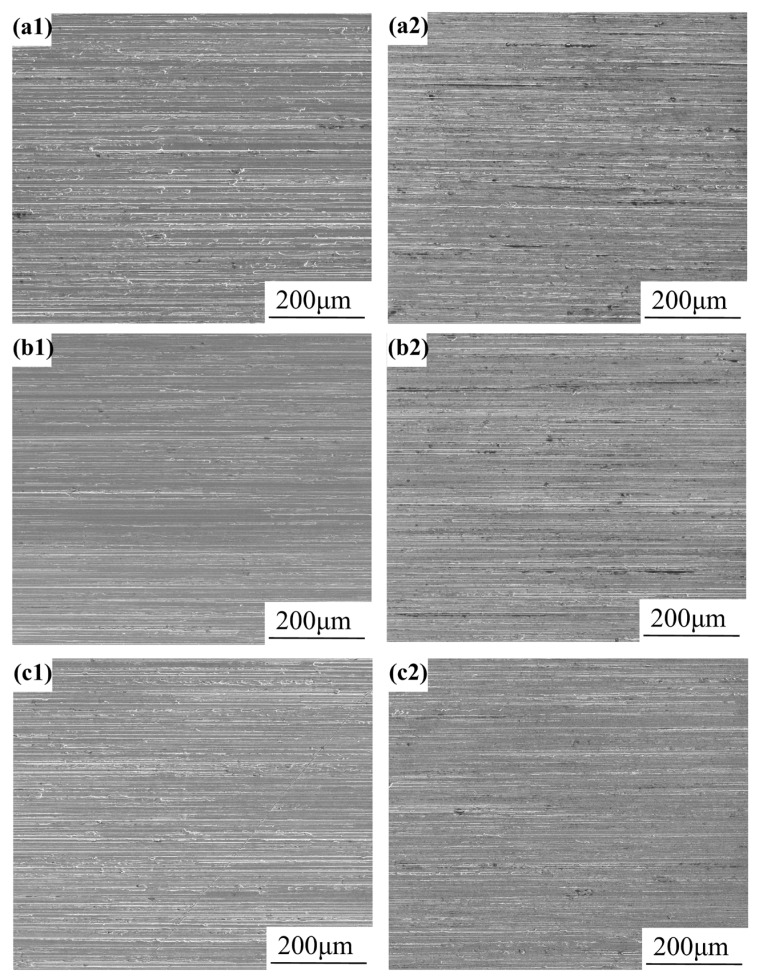
Microscopic corrosion morphology of immersed specimens in different Cl^−^ environments for 90 days: (**a1**) BM-100 mg/L; (**a2**) BM-300 mg/L; (**b1**) SMAW-100 mg/L; (**b2**) SMAW-300 mg/L; (**c1**) GMAW-100 mg/L; and (**c2**) GMAW-300 mg/L.

Electrochemical characterization guided the design of the immersion protocols simulating freshwater environments at 100 mg/L and 300 mg/L Cl^−^ concentrations. The 100 mg/L Cl^−^ condition simulates nominal service conditions, while 300 mg/L Cl^−^ represents an accelerated degradation scenario for the performance threshold evaluation. The macro- and micro-morphologies of specimens following 90 days of immersion are presented in Figure 8 and Figure 9, respectively. Corrosion rate measurements obtained from 90-day immersion tests were statistically analyzed and summarized in Table 4. Figure 8 and Figure 9 demonstrate that all immersed specimens maintained smooth surfaces without evidence of pitting corrosion. As quantified in Table 4, both the 304 stainless steel BM and welded joints exhibited negligible corrosion rates following the 90 days of immersion and subsequent descaling, demonstrating an exceptional resistance to general and localized corrosion.

#### 3.2.4. Stress Corrosion Performance Test Results

**Figure 10:**
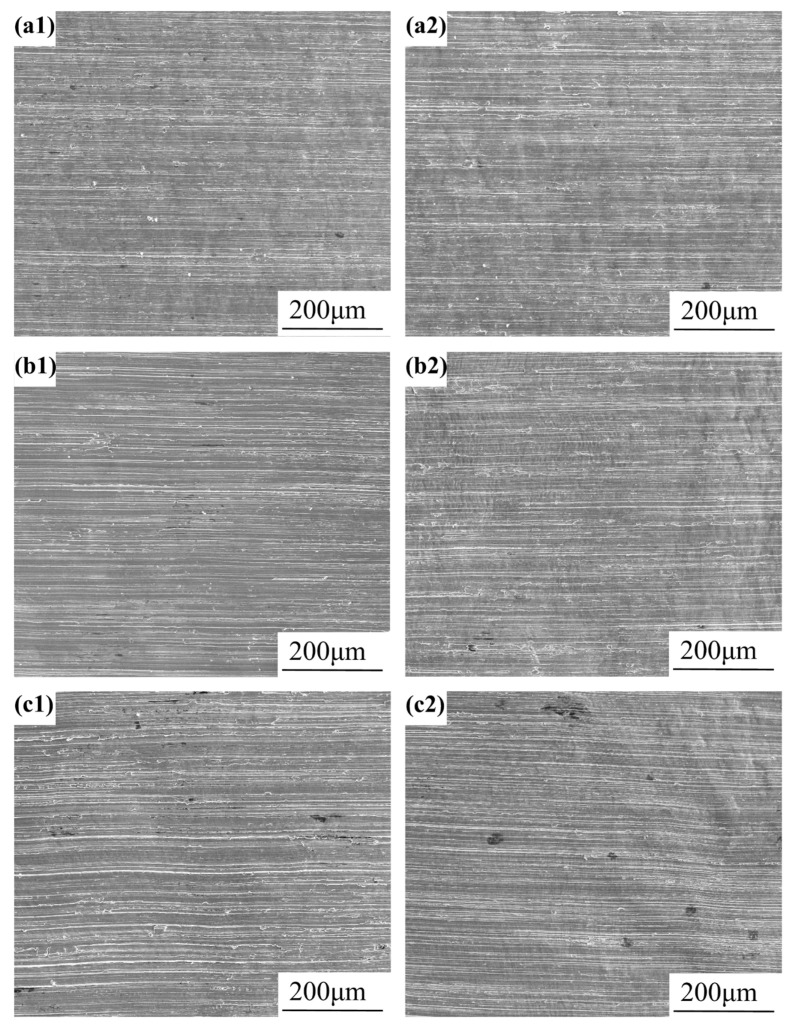
Microscopic corrosion morphology of U-bend specimen arc top area immersed in different Cl^−^ environments for 90 days: (**a1**) BM-100 mg/L; (**a2**) BM-300 mg/L; (**b1**) SMAW-100 mg/L; (**b2**) SMAW-300 mg/L; (**c1**) GMAW-100 mg/L; and (**c2**) GMAW-300 mg/L.

The micro-morphologies of the 304 stainless steel welded joint U-bend specimen arc top area subjected to different welding processes, following the immersion in a simulated water environment, are presented in Figure 10. The microstructural analysis of the immersed specimens reveals relatively smooth surfaces with preserved grinding marks and a complete absence of SCC even at a Cl^−^ concentration of 300 mg/L. These findings demonstrate that welded joints produced by both processes exhibit an excellent SCC resistance in chloride-containing environments.

## 4. Discussion

### 4.1. Corrosion Mechanism of 304 Stainless Steel Base Metal and Welded Joints in Environments with Different Cl^−^ Concentrations

The surface of the 304 stainless steel possesses a protective passive film that serves as a kinetic barrier against corrosion. When maintaining its integrity, the passive film effectively protects the underlying metal substrate against pitting initiation. [28] However, the film breakdown induced by aggressive species (e.g., Cl^−^ ions) or mechanical stress creates direct electrochemical pathways between the metal surface and corrosive environment, initiating localized corrosion. Through comprehensive electrochemical studies, Macdonald et al. [29] established the critical conditions for passive film breakdown, demonstrating that film dissolution initiates when the applied potential surpasses the critical pitting potential *V_c_*:

(3) $$V_{c}=A-B\log\alpha_{\chi }$$

where *A* and *B* are constants, and *α_χ_* denotes the activity of halide ions. As the Cl^−^ concentration in the electrolyte increases, the critical potential decreases, rendering the passive film increasingly susceptible to breakdown. This observation is corroborated by the polarization curve and electrochemical impedance spectroscopy analyses, demonstrating that elevated Cl^−^ concentrations enhance the corrosion propensity of both the 304 stainless steel matrix and welded joint structures. These findings align with established theories regarding chloride-induced localized corrosion mechanisms [30].

As established in prior studies [31,32], the anodic dissolution mechanism of 304 stainless steel substrates in near-neutral aqueous environments following passive film degradation can be described by

(4) $$Fe\to Fe^{2+}+2e^{-}$$

These Fe^2+^ ions subsequently react with hydroxide ions (OH^−^) from the water dissociation to form ferrous hydroxide:

(5) $$Fe^{2+}+2OH^{-}\to Fe{\left(OH\right)}_{2}$$

In an aqueous environment containing dissolved oxygen, Fe(OH)_2_ undergoes an oxidative transformation through the following pathways:

(6) $$2Fe{\left(OH\right)}_{2}+\frac{1}{2}O_{2}\to 2FeOOH+H_{2}O$$

(7) $$2FeOOH\to Fe_{2}O_{3}+H_{2}O$$

The electrochemical analysis of polarization curves in this work confirms oxygen reduction as the dominant cathodic process:

(8) $$O_{2}+4e^{-}+2H_{2}O\to 4OH^{-}$$

**Figure 11:**
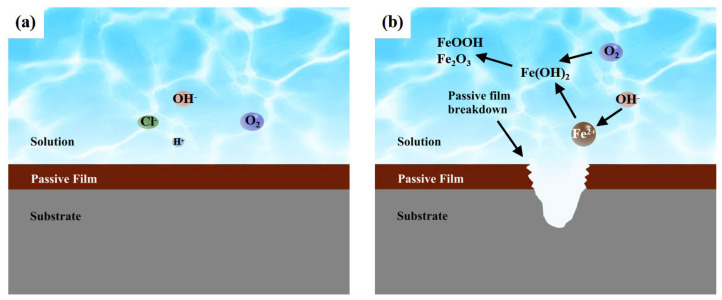
An illustration of the corrosion mechanism of the 304 stainless steel matrix and welded joints in aqueous solutions containing chloride ions. (**a**) The passivation film covers the surface of the substrate and prevents it from corrosion. (**b**) After the passivation film is destroyed, the substrate undergoes corrosive degradation in aqueous solutions containing chloride ions.

A graphical representation of the above reaction equation is shown in Figure 11. By applying the electrochemical stability criterion established by Macdonald et al. [29], our experimental data demonstrate that the chloride concentration (300 g/L Cl^−^) remains below the threshold required to depress the critical pitting potential (*E_pit_*) of both the 304 stainless steel BM and welded joints below their respective corrosion potentials. Differences in *i_p_* may arise from differences in the stability of passivation films formed in different regions of different welded joints, which may be attributed to microstructural differences and the degree of chromium depletion. Notably, SMAW-WM specimens displayed characteristic secondary passivation behavior at a 300 g/L Cl^−^ concentration, yet all tested materials maintained stable passive films with no measurable corrosion damage. This phenomenon in the WM region of the SMAW may be attributed to the relatively lower stability of the passivation film in the WM region [33] due to the residual stresses generated during the welding process [34]. Immersion testing in simulated freshwater environments (varying Cl^−^ concentrations, 90-day duration) revealed a negligible mass loss for both the BM and welded joint specimens, confirming their excellent corrosion resistance. This experimental consistency between the accelerated electrochemical measurements and long-term immersion performance validates the proposed corrosion resistance evaluation methodology.

The chromium depletion mechanism posits that intergranular corrosion in austenitic stainless steels originates from carbide precipitation at grain boundaries, resulting in localized Cr-depleted regions incapable of sustaining protective passive films [7]. These Cr-deficient zones fail to maintain the Cr-rich oxide layer required for passivity, triggering the electrochemical dissolution processes outlined in Reactions (4)–(8). This microstructural configuration establishes a galvanic couple where bulk grains (cathode) and Cr-depleted boundaries (anode) create large current densities, accelerating Reaction (4) kinetics as per the mixed-potential theory [32]. The DL-EPR testing revealed the low intergranular corrosion susceptibility (DOS < 1%) for both weldments, corroborated by the absence of intergranular corrosion in U-bend specimens after 90 days of immersion. These findings demonstrate an excellent intergranular corrosion resistance for both welding processes in investigated freshwater environments. The microstructural characterization (Figure 3a,b) showed a complete absence of the grain boundary precipitation of Cr and iron carbides, which indicates that both SMAW and GMAW processes effectively reduce carbide precipitation, maintaining boundary Cr levels above the passivation threshold. The comparative analysis showed a higher IGC susceptibility in the HAZ versus WM regions, attributed to differential thermal histories during welding. This phenomenon may correlate with the HAZ exhibiting a lower boundary Cr content than the WM, resulting in the formation of unstable or defective passive films that are more prone to failure.

### 4.2. The Effect of the Welding Process on the Corrosion of the 304 Stainless Steel Base Metal and Welded Joints

The comparative analysis of the electrochemical and immersion test data for SMAW and GMAW weldments reveals a comparable corrosion resistance in simulated freshwater environments. Observed variations in the electrochemical response between the weldments originate from distinct microstructural characteristics developed during each welding process. The metallographic analysis (Figure 3) indicates a higher ferrite content in the GMAW metal compared to its SMAW counterparts. The observed microstructural divergence suggests differential thermal inputs between SMAW (manual) and GMAW processes. As documented in Ref. [15], an elevated thermal input decreases the weld ferrite content and consequently degrades the passivation capacity. Therefore, the higher ferrite content in the gas shielded welded joint structure may indicate a lower thermal input during the welding process, which results in a stronger passivation ability at elevated Cl^−^ concentrations (300 mg/L). However, at lower Cl^−^ concentrations (50–150 mg/L), SMAW joints unexpectedly demonstrated a lower corrosion current density than GMAW specimens, contrasting with prior findings [14]. The reasons for this require further study.

The DL-EPR testing revealed comparable degrees of sensitization for both weldments, confirming the welding process’s irrelevance with the intergranular corrosion resistance in 304 stainless steel joints under simulated service conditions.

### 4.3. The Effect of Stress on the 304 Stainless Steel BM and Welded Joints

The synergistic interaction between the applied stress and chloride-containing environments also affects the corrosion behavior of 304 stainless steel degradation mechanisms of the 304 stainless steel BM and weldments [27,35]. Mechanical stress concentrations exceeding the cohesive strength of passive films induce localized film rupture, creating active dissolution sites where Reactions (4)–(8) propagate and lead to corrosion [36]. The reaction process results in a stress concentration at the corrosion defects, ultimately causing the nucleation of microcracks. In this study, after immersing U-bend samples of the 304 stainless steel BM and different welded joints in a simulated freshwater environment for 90 days, the surfaces remained bright, and the SEM characterization showed preserved machining marks and a complete absence of SCC initiation sites. These findings demonstrate stable passivation states under sustained stress conditions, confirming that the environmental stress conditions in simulated the freshwater environment (<300 mg/L Cl^−^) remain below critical thresholds for passive film breakdown in both the BM and weldments.

In summary, both the 304 stainless steel BM and SMAW/GMAW weldments prepared by different welding processes exhibit a good corrosion resistance in the simulated freshwater environment, demonstrating excellent environmental adaptability. The relationship between the content of ferrite in the organization of welded joints and the corrosion resistance is not the same as previous studies, which requires further experimental verification. In addition, although neither the stainless steel base material nor the welded joints corroded in the immersion test and showed an excellent corrosion resistance, whether the difference in the corrosion resistance will be reflected in a longer immersion test cycle is also the next research direction of this study.

## 5. Conclusions

In the simulated freshwater environment, the corrosion behavior of the 304 stainless steel base metal and welded joints was studied, and the conclusions are as follows:

(1) The effect of chloride ions on the corrosion behavior of the 304 stainless steel substrate and welded joints is consistent at a concentration range of 50~300 mg/L. With the increase in the Cl^−^ concentration, the corrosion resistance of the 304 stainless steel base metal and welded joints decreases.
(2) All weldments showed a low intergranular corrosion (below 1%) susceptibility in the tested environment, with the HAZ exhibiting a higher DOS (0.196%, 0.0832%) than WM regions (0.4716%, 0.781). The DOS of the HAZ of the GMAW welded joints is about twice as much as that of the SMAW welded joints.
(3) Both the BM and welded joints of the 304 stainless steel have a good corrosion resistance. The presence of different welding processes and stresses had little effect on the corrosion resistance of 304 stainless steel welded joints during the long-term immersion test.

## Figures and Tables

**Table 1:** The chemical compositions of the welding feeding wire (wt.%).

	C	Si	Mn	P	S	Cr	Ni	Mo	Cu
A102	0.08	1.00	0.5–2.5	0.04	0.03	18.0–21.0	9.0–11.0	0.75	0.75
ER308L	0.03	0.60	1.80	0.015	0.008	20.0	10.0	-	-

**Table 2:** Simulated solution composition for water environment of reservoir environment with different Cl^−^ concentrations (mg/L).

	CaCl_2_	MgCl_2_	KCl	NaHCO_3_	Na_2_SO_4_	NaCl
50	29.31	37.99	6.00	262.54	193.31	/
100	29.31	37.99	6.00	262.54	192.31	82.39
150	29.31	37.99	6.00	262.54	192.31	165.67
300	29.31	37.99	6.00	262.54	192.31	412.95

**Table 3:** Sensitivity of IGC in 304 stainless steel welded specimens.

Welding Method	Microstructure	DOS (%)
304-SMAW	WM	0.196
	HAZ	0.4716
304-GMAW	WM	0.0832
	HAZ	0.781

**Table 4:** Corrosion rate of different 304 welding specimens at different Cl^−^ concentrations.

Cl^−^ Concentration	BM Corrosion Rate (mm/a)	SMAW Corrosion Rate (mm/a)	GMAW Corrosion Rate (mm/a)
100 mg/L	0.00	0.00	0.00
300 mg/L	1.13 × 10^−3^	0.00	0.00

## Data Availability

The original contributions presented in this study are included in the article. Further inquiries can be directed to the corresponding author.
